# Letter from London—Letters on Horseback

**Published:** 1878-07

**Authors:** 


### LETTER FROM LONDON.

LONDON, ENG., June 1, 1878.

Johnny is an out-door patient of the Victoria Hospital for children, Site street, Queen's Road, London. It was through him I gained an introduction to the lady superintendent, Miss Hamilton, who kindly showed us over the building. It is called Gough House, and once belonged to a lord of that name. They say that Lady Gough was murdered there by her husband, and that ever since, her spirit has haunted the underground passages which lead to the river Thames. Surely not a very pleasant fancy for childish minds to dwell upon.

The Hospital contains some sixty children, of ages ranging between two years and thirteen. The wards are named after their respective patrons. Princess Louise, Princess Alice, Lord Cadogan, Harrie Faraquhar, Esq., and others.

We went on visitors day, when the children's mothers and relations came to see them. There was quite a crowd about the entrance, mothers, brothers, sisters, and friends, giving their little invalids a breath of fresh air. One among them was a girl about eleven years old, who was carrying her crippled brother, a great boy of nine, up and down the garden walk, singing bits of nursery rhyme, and trying to dissipate by her kindly voice and gentle manner, the remembrance of pain and suffering.

The children appeared far happier than I expected they would be. Some were playing ball, some pasting pictures into big scrap-books, some jingling on a piano, and all apparently having a good time. At first sight I would have thought them healthy and happy; but a second glance showed them all more or less pale, with great blue circles around their childish eyes—and some little hands were too weak to hold the beautiful picture books.

One boy I noticed particularly. He was propped up by pillows, and held in his listless hand an orange some one had given him. He gazed straight in front of him, unconscious of the noise about—deep in his own misery, lonely, mother-sick. Suddenly I saw his face change, his lips quivered, his arms stretched out, and with a cry of "Mamma!" he threw himself upon the breast of a red-faced, motherly woman who had just entered, and sobbed aloud. "Abscess in the neck, very painful," said Miss Hamilton, and we passed on.

Near the window, which over-looked a small garden, and the numberless gray house-tops of smoky London, lay another little fellow, hopelessly crippled. By his bed-side sat a broad-shouldered boy, with red healthy cheeks, and restless feet. He was reading Hans Anderson's tales aloud, and they were both laughing heartily. Miss Hamilton said the red cheeked boy came every Thursday, (visitors day), as early as the doors were opened and stayed till they were closed. He had never missed a day since the lame boy came to the hospital, rainy or snowy, or what is more for a hearty fellow like him, warm, sunshiny spring days never tempted him from his post; he was always there, with a new book and long school boy stories.

I sat down by the bedside of a little girl and asked her why she looked so happy. She told me she was going the next day to Margate where they send the convalescent children.

"Do you like it here?" I asked.

"I don't mind it in the daytime," she said, "but at night I don't like to hear the other children crying."

"The nurses look very kind," I said, noticing a young lady dancing and singing "four-and-twenty black birds," for the amusement of half a dozen baby invalids, who were sitting up in their beds, and watching her with great interest and delight. "They are kind," said my little girl, her face lighting up; and if you are going to write about this place, *do* mention Miss Turner. She's a day nurse, you know, but she sat up all night with me once, just because I was frightened, and never said a word about being tired. Do write something about that, and," she added, showing herself truly feminine, "Say she's very pretty, too."

A woman sat near, rocking her baby to and fro, and trying to hide the big tears which rolled down her cheeks. She called to Miss Hamilton, "When baby cries this way," she said anxiously, "he wants a drink of water. You'll remember, won't you? Truly, truly you'll remember?"

"O, yes, indeed," was the answer, "I know all that."

"Are you married? Have you any children of your own?" asked the woman again.

Miss Hamilton smiled and shook her head.

"Then you cannot take care of my baby! Why don't they have married women here, mothers who know what it is to have a child ill and dying!"

She must have seen the compassion in my face, for she turned to me and told her story. She was a poor woman, she said, and gave her child out to nurse while she worked to support it. The nurse had charge of two other children, one of whom is dead, the other not expected to live. The drains were bad, and the nurse did not tell the mother for fear of losing money.

"I took him to the parish doctor," said the woman, holding her baby close and looking lovingly down into its pinched white face, "but he would do nothing, as it was ill through carelessness, and so I had to bring him here, leave him in care of strangers, and I only see him once a week. "Ah, well," she added with a sigh, "he's better off here than he would be at home."

Then Miss Hamilton tried to interest me by giving a long account of their trial of Dr. Sayre's Plaster of Paris invention, and how well it had succeeded. How one miserable child, only eight years old, had been there nine months for dropsy, and had been tapped eighteen times. How they taught women to be nurses there. She would have gone on forever, I suppose, but I could bear no more. I thanked her kindly, took one last look along the row of little white beds, and the whiter faces, with their sad, weary eyes, and givmy hand to Johnny, we went away.

ISOBEL OSBOURNE.

---

It may not be a modest act to give publicity to the following, but at the urgent request of the young gentlemen whose names appear below, we shut our eyes, and let the BISTOURY have it:

OFFICE OF DR. J. W. SHEETS,
SELINSGROVE, Pa., April 12, 1878.

At a meeting of the students under Dr. J. W. Sheets, the following resolutions were unanimously adopted:

*Resolved*, That we, as Medical Students, are under many obligations to Dr. Thad S. Up de-Graff, of Elmira, N. Y., for the very able and interesting lecture he delivered in our place, April 8th, 1878.

*Resolved*, That we return our hearty thanks to him for permitting us to witness the skillful operations he performed for Cataract, Strabismus, Pterygium, &c.

*Resolved*, That we have been greatly benefited by his visit to his native home, and that we shall ever remember Dr. Up de Graff as a skilled surgeon, and for his many generous acts while in our midst.

On motion, it was agreed that the above resolutions be put on file, and a copy of the same be sent Dr. Up de Graff.

P. A. BOYN, B. F. EMRICK,
MARION ULRICH, H. M. EMRICK,
T. J. HILBISH, D. H. LOCKARD,
Students from Shamokin.

COLUMBUS, OHIO, June 1, 1878.

DR. UP DE GRAFF:

I read that horseback article of yours, got a horse and bridle—likewise saddle, and rode twenty miles yesterday. I was charmed with the ride, delighted with the exercise, but up to this writing I have not been out of bed. I think I read somewhere in the BISTOURY that brandy and brown paper was good for the raw places. Did you ever try that prescription yourself? Goodness, how it does smart! If I live, I don't think I'll follow up your cure.

Wretchedly thine, JOHN.

Well, John, you simply overdid the matter. Try again, and ride but one mile next time, increasing the distance daily. You will soon be able to take your twenty mile ride without discomfort, and no use for the b. & b. p.

---

WILKESBARRE, PA., May 20, 1878.

DR. UP DE GRAFF:

That Bistoury article on horseback-riding captured me—I've been at it ever since—it's bully. Tell me something more to do that's as good.

L. M.

Go fishing.

---

HANNIBAL, MO., June 5, 1878.

DR. THAD S. UP DE GRAFF:

I do wonder how many poor devils you have killed with that horseback article of yours? Dear O me! I haven't got a perfectly well spot anywhere on my whole body. But then, I may have overdone the matter—I rode sixty miles two days ago. What new invention will you have for us miserable dispeptics next time? I wait and tremble.

B.

Camp out—try it.

---

CANANDAIGUA, N. Y., June 25, 1878.

DR. UP DE GRAFF:

See here, Mr. Doctor, you just write any more nonsense like that horseback affair of yours and some one will get killed—oh!

W.

There's another chap overdid the business.

---

BINGHAMTON, N. Y., June 25, 1878.

EDITOR BISTOURY:

That horseback article did the business for me. Dyspepsia all gone. Ride every morning, good times, glorious! T. L. B.

Good boy. Keep it up.

---

WILLIAMSPORT, PA., May 15, 1878.

DR. UP DE GRAFF:

Come down and skin graft a place as big as a saucer on each side. I tried that horseback prescription. Thine, B.

Put a plaster on it and try again. Nothing like it.

